# On the Intermaxillary Bone and Its Relations to Hare-Lip and Cleft Palate

**Published:** 1862-03

**Authors:** 


On the Intermaxillary Bone and its Relations to Hare-lip and Cleft Palate.—The Bonn correspondent of the *Medical Times and Gazette* gives the following summary of a paper on this subject, read by Prof. C. O. Weber before the Scientific Association of the Rhenish Provinces and Westphalia. It "was illustrated," he says, "by a beautiful series of preparations, showing the different degrees of the incomplete union of the lip and the palate. The author introduced his subject by stating that the great German poet Goëthe had been the first to show the existence of the intermaxillary bone in man. This view had at first been very much contradicted, but it was now universally adopted, and it was, in fact, easy to show this bone in the skull of a newly-born infant. It was this bone, the development of which was of so great an importance for the shape of the upper lip and the palate. The natural cleft lip was the normal formation in the hare, the camel, and the lamb, while in man it was exceedingly rare, and only connected with the complete deficiency of the intermaxillary bone and the soft part corresponding to it, viz.: the filtrum. The terms hare-lip and the wolf's gullet (as cleft palate is called in Germany) were, therefore, not correct, as the locality of the malformation was generally different.

The evolution of the mouth and the jaw in the human fœtus was then described. In the second week of fœtal life severel lobes grew up at both sides of the head below the brain-vesicles; these are the so-called branchiate arches or facial lobes, of which the two upper ones correspond to the upper jaw, the second to the tongue, the third to the lower jaw, and the fourth to the hyoid bone. The three lower ones unite, while between the lobes of the upper and the lower jaw a cleft remains, which forms the mouth. The frontal lobe, from which the nose, the middle part of the lip, and the intermaxillary bone are formed, grows afterward between the two lobes of the upper jaw. The anterior portion of the lobes is, in the course of time, changed into soft parts, the posterior one into bone. The development of these parts is independent of each other, and, therefore, a deficient union of soft and of osseous parts may exist, the one without the other, although they are often found combined. If the evolution is impeded at an early period, the growth of the parts in another direction is not prevented thereby; and according as the impediment occurs early or late, and is complete or incomplete, a great variety of malformations is the result. The highest degree of it would be double facial and palatal cleft, if the frontal lobe did not unite at all with the upper maxillary lobe, and were further developed without reference to the latter. Such complete clefts, however, have, until now, only been observed on one side. If the upper part unites with the frontal lobe, while there is a unilateral cleft in the lower one, we have unilateral hare-lip and cleft palate; the hare-lip then leads through the corresponding nostril into the cleft palate.

If the deficient union of the frontal lobe with the upper maxillary lobe only affects the soft parts anteriorly, there is unilateral or double hare-lip of different extent; and if the soft portions of the face unite, the osseous parts remaining apart, there are clefts of the palate and of the jaw.

"The causes of the origin of this abnormal evolution in the fœtus are still involved in much obscurity. It is true that in some cases the affection has appeared hereditary, but this is by no means the rule. An influence of the imagination of the mother upon the evolution of the embryo, which is believed to be the cause by the people, can not, *a priori*, be denied, as the influence of the mind on nutrition is evident to every one. Most of the cases, however, which are mentioned in support of this hypothesis can not be accepted as proofs, as in them the mind of the mother was only affected long after the defective evolution had commenced. As these clefts of the lip and palate are to be distinguished after the sixth week, fright of the mother could only cause them if it occurred during the first weeks of pregnancy. Professor Weber showed a preparation of a fœtus of three months, with total cleft of the lip and the palate."

---

In the course of some remarks on the physiological relations of sugar, a contemporary thus refers to certain facts connected with its supposed influence upon the teeth: "Sugar lies under a ban for injuring the teeth; but the negroes employed on sugar plantations, who eat, perhaps, more sugar than any other class of people, have almost proverbially fine, white, sound teeth, which they retain in old age. But, on the other hand, in England, persons employed in the sugar refineries, who are, from their occupation, obliged constantly to be tasting sugar, lose their teeth from decay after a few years. Sugar in combination with a small amount of alkali has the property of dissolving phosphate of lime, which is contained in large quantities in the bone and teeth; a circumstance which may explain in some measure the contradictory nature of the facts."

